# Transthoracic Versus Transhiatal Esophagectomy for Esophageal Cancer: A Nationwide Propensity Score-Matched Cohort Analysis

**DOI:** 10.1245/s10434-020-08760-8

**Published:** 2020-06-30

**Authors:** Alexander C. Mertens, Marianne C. Kalff, Wietse J. Eshuis, Thomas M. Van Gulik, Mark I. Van Berge Henegouwen, Suzanne S. Gisbertz, the Dutch Upper GI Cancer Audit group

**Affiliations:** 1 Amsterdam UMC, Department of Surgery, Cancer Center Amsterdam, University of Amsterdam, Amsterdam, The Netherlands; 2 Robotics and Mechatronics, University of Twente, Enschede, The Netherlands

**Keywords:** Upper gastrointestinal tract, Neoplasms, Esophagectomy, Transhiatal, Transthoracic

## Abstract

**Background:**

Chemoradiation followed by resection has been the standard therapy for resectable (cT1-4aN0-3M0) esophageal carcinoma in the Netherlands since 2010. The optimal surgical approach remains a matter of debate. Therefore, the purpose of this study was to compare the transhiatal and the transthoracic approach concerning morbidity, mortality and oncological quality.

**Methods:**

Data was acquired from the Dutch Upper GI Cancer Audit. Patients who underwent esophagectomy with curative intent and gastric tube reconstruction for mid/distal esophageal or esophagogastric junction carcinoma (cT1-4aN0-3M0) from 2011 to 2016 were included. Patients who underwent a transthoracic and transhiatal esophagectomy were compared after propensity score matching.

**Results:**

After propensity score matching, 1532 of 4143 patients were included for analysis. The transthoracic approach yielded more lymph nodes (transthoracic median 19, transhiatal median 14; *p* < 0.001). There was no difference in the number of positive lymph nodes, however, the median (y)pN-stage was higher in the transthoracic group (*p* = 0.044). The transthoracic group experienced more chyle leakage (9.7% vs. 2.7%, *p* < 0.001), more pulmonary complications (35.5% vs. 26.1%, *p *< 0.001), and more cardiac complications (15.4% vs. 10.3%, *p* = 0.003). The transthoracic group required a longer hospital stay (median 14 vs. 11 days, *p *< 0.001), ICU stay (median 3 vs. 1 day, *p* < 0.001), and had a higher 30-day/in-hospital mortality rate (4.0% vs. 1.7%, *p* = 0.009).

**Conclusions:**

In a propensity score-matched cohort, the transthoracic esophagectomy provided a more extensive lymph node dissection, which resulted in a higher lymph node yield, at the cost of increased morbidity and short-term mortality.

As of 2012, esophageal cancer is the eighth most common malignancy worldwide. Both globally and in the Netherlands, a trend of increasing incidence and mortality has been reported.^1^^,^^2^ Neoadjuvant chemoradiation (nCRT) following the CROSS regimen with subsequent resection has been the standard treatment for resectable (cT2-4aN0-3M0 and T0-1 N + M0) esophageal carcinoma in the Netherlands since 2010.^3^ While neoadjuvant treatment is fairly standardized in the Netherlands, the optimal surgical approach remains a matter of active debate in both literature and daily practice.

The largest randomized, controlled trial comparing a transthoracic with a transhiatal approach dates back to 2002.^4^^,^^5^ It illustrated a trend towards improved survival for patients after a transthoracic resection, in conjunction with a significant 5-year overall survival benefit for the subgroup of patients with 1-8 positive nodes in the resection specimen.^4^,^5^ However, this trial predates neoadjuvant therapy and is restricted to open procedures, possibly making these results less applicable to current practice. The latest meta-analysis on this subject was published in 2011 and did not find a difference in survival.^6^ It did, however, describe a higher short-term mortality, longer hospital stay, higher lymph node yield, and lower anastomotic leakage rate in the transthoracic group.

Therefore, the purpose of the current study was to compare the transhiatal and transthoracic approach regarding morbidity, mortality, and the quality of the surgical resection for resectable lower esophageal and junction carcinoma in a nationwide cohort study in the Netherlands.

## Methods

Data were obtained from the Dutch Upper GI Cancer Audit (DUCA). This audit was initiated in 2011 and is part of the Dutch Institute for Clinical Auditing (DICA). In the Netherlands, caregivers are obligated to register all patients with esophageal or gastric cancer with intended resection in the DUCA database. The main goal of this audit was to provide transparent information on the quality of care. Validation of completeness and accuracy of data registration is performed as has been described in earlier publications.^7^ Because the audit data are available anonymously, it is not possible to retrospectively retrieve missing data or include variables, such as surgical procedural data, hospital of treatment, 90-day mortality, or survival, outside the scope of the audit. This study was approved by the scientific committee of the DUCA. No informed consent or ethical approval was required under Dutch law. All procedures followed were in accordance with the ethical standards of the responsible committee on human experimentation (institutional and national) and with the Helsinki Declaration of 1975 and later versions.

### Patient Characteristics and Clinical Data

All patients undergoing surgery with curative intent for mid to distal esophageal or junction carcinoma (cT1-4aN0-3M0), including cTxNx, from 2011 through 2016 were retrieved from the database. Patients undergoing a three-stage McKeown (cervical anastomosis), a two-stage Ivor Lewis (thoracic anastomosis), or a transhiatal (cervical anastomosis) procedure with gastric tube reconstruction were included. Patients with missing baseline data and patients undergoing emergency surgery were excluded. Patients undergoing a hybrid resection were excluded due to the heterogeneity of this group; there was no possibility to discern between a laparoscopy combined with a thoracotomy or a laparotomy combined with thoracoscopy.

### Outcome Data

The main outcomes were quality-indicators of the surgical resection specimen, including R0 resection rate, circumferential resection margin, and lymph node yield.

Patient, tumor, and treatment characteristics, including perioperative and pathological outcomes, were retrieved from the DUCA database. Complications were defined according to standards of the DUCA. Anastomotic leakage was defined as a clinically or radiologically diagnosed leakage of the esophagus, stomach, anastomosis, or staple line, independent of presentation. Recurrent nerve lesions were scored without severity, because this was not reported consistently. Short-term oncologic and clinical outcomes were analyzed, including lymph node yield and radicality of resection. Because the DUCA only registers outcomes during the hospital stay and at least the first 30 postoperative days, long-term outcomes were not available for analysis. In-hospital and 30-day mortality are a combined item in the DUCA registration. The Clavien-Dindo classification for complications was only recently added to the audit and omitted from the analysis because of missing data in the earlier years.

### Statistical Analysis

The study population was divided into two groups: TTE and THE. To minimize the effect of confounders on the outcomes between these groups, a propensity score-matching analysis was performed. A propensity score was calculated for each patient through logistic regression, based on all covariates (*n* = 15) displayed as baseline characteristics in Table 1. Using nearest-neighbor matching without replacement, matched pairs of cases were identified. A caliper of 0.2 was set to prevent poor matches. The balance of the matched cohort was assessed using the standardized mean difference (SMD). A SMD < 10% was taken to indicate sufficient balance.

**Table 1:** Baseline data comparing the unmatched to the propensity matched cohort, with subdivision between transthoracic and transhiatal approach

Variable	Unmatched cohort					After propensity score matching				
	TTE (*N* = 2409)		THE (*N* = 1198)			TTE (*N* = 766)		THE (*N* = 766)		
	*N*	%	*N*	%	SMD	*N*	%	*N*	%	SMD
Sex										
Female	555	23.0	243	20.3	0.057	145	18.9	154	20.1	0.030
Male	1854	77.0	955	79.7		621	81.1	612	79.9	
Age *median*	65	[59–70]	66	[60–72]	0.193	66	[21–71]	66	[60–72]	0.026
BMI *median*	25	[23–28]	26	[23–29]	0.124	26	[23–28]	26	[23–29]	0.003
ASA-score										
I	419	17.4	192	16.0	0.151	122	15.9	120	15.7	0.065
II	1506	62.5	694	57.9		445	58.1	462	60.3	
III	478	19.8	303	25.3		196	25.6	179	23.4	
IV	6	0.2	9	0.8		3	0.4	5	0.7	
Comorbidities										
Pulmonary	421	17.5	242	20.2	0.070	143	18.7	148	19.3	0.017
Cardiac	528	21.9	312	26.0	0.097	190	24.8	195	25.5	0.015
Vascular	866	35.9	508	42.4	0.133	295	38.5	308	40.2	0.035
Diabetes	332	13.8	219	18.3	0.123	138	18.0	133	17.4	0.017
Histology										
AC	1841	76.4	1055	88.1	0.308	651	85.0	651	85.0	0.001
SCC	568	23.6	143	11.9		115	15.0	115	15.0	
cT stage										
Tis	3	0.1	1	0.1	0.039	2	0.3	1	0.1	0.057
T1	122	5.1	58	4.8		48	6.3	43	5.6	
T2	439	18.2	234	19.5		140	18.3	141	18.4	
T3	1684	69.9	828	69.1		517	67.5	529	69.1	
T4	71	2.9	32	2.7		26	3.4	22	2.9	
Tx	90	3.7	45	3.8		33	4.3	30	3.9	
cN stage										
*N*0	818	34.0	445	37.1	0.113	265	34.6	292	38.1	0.100
*N*1	988	41.0	473	39.5		300	39.2	288	37.6	
*N*2	448	18.6	201	16.8		143	18.7	141	18.4	
*N*3	74	3.1	26	2.2		20	2.6	18	2.3	
*N*+	25	1.0	12	1.0		9	1.2	8	1.0	
*N*x	56	2.3	41	3.4		29	3.8	19	2.5	
Location of tumor										
Middle	410	17.0	31	2.6	0.644	28	3.7	31	4.0	0.029
Distal	1598	66.3	721	60.2		521	68.0	512	66.8	
GEJ	401	16.6	446	37.2		217	28.3	223	29.1	
Neoadjuvant treatment										
None	163	6.8	124	10.4	0.190	78	10.2	81	10.6	0.044
Chemother.	130	5.4	103	8.6		66	8.6	57	7.4	
CRT	2115	87.8	971	81.1		622	81.2	628	82.0	
Year of surgery										
2011	226	9.4	271	22.6	0.508	120	15.7	122	15.9	0.038
2012	348	14.4	231	19.3		142	18.5	135	17.6	
2013	348	14.4	195	16.3		123	16.1	125	16.3	
2014	439	18.2	198	16.5		138	18.0	144	18.8	
2015	524	21.8	189	15.8		136	17.8	139	18.1	
2016	524	21.8	114	9.5		107	14.0	101	13.2	
Type of surgery										
MIS	1836	76.2	323	27.0	1.110	321	41.9	323	42.2	0.005
Open	573	23.8	866	72.3		445	58.1	443	57.8	

Percentages might not add up to 100% due to rounding. Numbers between brackets depict the interquartile range. *AC* adenocarcinoma; *ASA* American Society of Anesthesiologists; *BMI* body mass index; *cT* clinical T stage; *cN* clinical N stage; *CRT* chemoradiotherapy; *GEJ* gastroesophageal junction; *MIS* minimally invasive surgery; *SCC* squamous cell carcinoma; *SMD* standardized mean difference; *TTE* transthoracic esophagectomy; *THE* transhiatal esophagectomy

The open-source software R 3.5.1 with packages “Matching” version 4.9-3 was used in the propensity score matching process.^8^^,^^9^ After assessing balance, the matched cohort was exported for use with SPSS Statistics Version 25.0 (Armonk, NY) for further statistical analysis. Evaluation of differences in outcomes between the two groups after matching was done by using paired tests:^10^ Paired Student’s *t* test for continuous parametric variables, Wilcoxon signed-rank test for nonparametric continuous or ordinal variables and McNemar’s test for nominal variables. Minimally invasive procedures converted to open surgery were analyzed as minimally invasive procedures. All hypothesis tests were two-sided. *P* values < 0.05 were considered statistically significant.

## Results

### Study Population

From 2011 through 2016, 4143 patients underwent an esophagectomy with curative intent in the Netherlands. In total, 536 (13%) patients were excluded from further analysis due to nonelective surgery (*n* = 13), cervical esophageal carcinoma (*n* = 44), reconstruction other than gastric tube (*n* = 64), hybrid surgery (*n* = 114), or missing preoperative data (*n* = 301).

Patients were divided into two groups based on the operative approach: transthoracic (TTE) or transhiatal (THE) esophagectomy. As depicted in Table 1, 11 of 15 baseline characteristics were unequally distributed between the groups in the unmatched cohort (SMD > 0.10). Through propensity score matching, 766 patients were matched in each group. The matched cohort was well balanced.

### Surgical and Histopathological Outcomes

Surgical and histopathological outcomes are shown in Table 2. In the propensity score matched cohort, the transthoracic approach yielded more lymph nodes (TTE median 19, interquartile range [IQR] 15–26; THE median 14, IQR 10–19; *p* < 0.001), but there was no difference in the median number of positive lymph nodes. Additionally, the TTE group had a higher (y)pN stage, even though the groups were matched on cN stage. The (y)pT stage, (y)pM stage, and the response to neoadjuvant therapy were distributed equally between groups. R0 resection was achieved in 94% of cases (TTE 93.9%, THE 93.6%). Due to the nature of the two surgical procedures, all patients in the THE group had a cervical anastomosis, whereas the TTE group contained both cervical and intrathoracic anastomoses. The distribution of the anastomotic location was comparable to the unmatched cohort.

**Table 2:** surgical and histopathological outcomes, showing both the unmatched and the propensity matched cohort, with comparison between transthoracic and transhiatal approach

Variable	Unmatched cohort					After propensity score matching				
	TTE (*N* = 2409)		THE (*N* = 1198)			TTE (*N* = 766)		THE (*N* = 766)		
	*N*	%	*N*	%	*P*	*N*	%	*N*	%	*P*
Anastomosis										
Cervical	1218	50.6	1198	100.0	< 0.001	376	49.1	766	100.0	< 0.001
Intrathoracic	1191	49.4	–	–		390	50.9	0	0.0	
Conversion^a^										
None	1727	71.7	297	24.8	< 0.001	302	39.4	288	37.6	0.728
Early	18	0.7	7	0.6		4	0.5	7	0.9	
Late	43	1.8	10	0.8		6	0.8	10	1.3	
NA (open)	573	23.8	866	72.3		445	58.1	443	57.8	
Resection										
R0	2266	94.1	1116	93.2	0.012	719	93.9	717	93.6	0.109
R1	115	4.8	65	5.4		44	5.7	39	5.1	
R2	0	0.0	4	0.3		0	0.0	2	0.3	
CRM *median, mm*	3	[1–7]	2.5	[1–6]	0.004	3	[1–6]	3	[2–7]	0.549
Lymph nodes, *median*										
Number	20	[15–27]	14	[10–19]	< 0.001	19	[15–26]	14	[10–19]	< 0.001
Positive	0	[0–1]	0	[0–2]	0.560	0	[0–2]	0	[0–2]	0.375
Ratio	0	[0–0.07]	0	[0–0.13]	0.030	0	[0–0.09]	0	[0–0.12]	0.122
(y)pT stage										
T0	553	23.0	256	21.4	0.038	150	19.6	164	21.4	0.404
Tis	18	0.7	15	1.3		8	1.0	10	1.3	
T1	416	17.3	169	14.1		123	16.1	116	15.1	
T2	432	17.9	245	20.5		131	17.1	154	20.1	
T3	850	35.3	448	37.4		311	40.6	280	36.6	
T4	6	0.2	4	0.3		5	0.7	8	1.0	
Tx	11	0.5	10	0.8		38	5.0	34	4.4	
(y)pN stage										
*N*0	1380	57.3	713	59.5	0.165	426	55.6	461	60.2	0.044
*N*1	516	21.4	221	18.4		172	22.5	151	19.7	
*N*2	262	10.9	144	12.0		87	11.4	79	10.3	
*N*3	138	5.7	67	5.6		46	6.0	41	5.4	
*N*x	2	0.1	6	0.5		35	4.6	34	4.4	
(y)pM stage										
M0	2300	95.5	1164	97.2	0.374	729	95.2	739	96.5	0.557
M1	22	0.9	15	1.3		7	0.9	12	1.6	
Mx	54	2.2	6	0.5		30	3.9	15	2.0	
Response to neoadjuvant treatment										
None	214	8.9	85	7.1	< 0.001	73	9.5	47	6.1	0.073
Partial	1267	52.6	632	52.8		390	50.9	405	52.9	
Complete	659	27.4	307	25.6		190	24.8	204	26.6	

Percentages might not add up to 100% due to rounding. Numbers between brackets depict the interquartile range. *mm* millimeters; *NA* not applicable; *TTE* transthoracic esophagectomy; *THE* transhiatal esophagectomy; *(y)pT* pathological T stage; *(y)pN* pathological N stage; *(y)pM* pathological M stage

^a^Early conversion < 30 min of incision, late conversion > 30 min of incision

### Postoperative Outcomes

Table 3 depicts the postoperative outcomes. After propensity score matching, no statistically significant differences remained in morbidity (62.9% vs. 58.2%, *p* = 0.054) and infections (*p* = 0.099). The TTE group less often received tube feeding (86.2% vs. 93.0%, *p* < 0.001), experienced more chyle leakages (9.7% vs. 2.7%, *p* < 0.001), more pulmonary complications (35.5% vs. 26.1%, *p* < 0.001), and more cardiac complications (15.4% vs. 10.3%, *p* = 0.003). In addition to this, the TTE group had a longer hospital stay (median 14 vs. 11 days, *p* < 0.001), longer ICU stay (median 3 vs. 1 day, *p* < 0.001), and had a higher in-hospital/30-day mortality (4.0% vs. 1.7%, *p* = 0.009).

**Table 3:** Postoperative outcomes, showing both the unmatched and the propensity-matched cohort, with comparison between transthoracic and transhiatal approach

Variable	Unmatched cohort					After propensity score matching				
	TTE (N = 2409)		THE (N = 1198)			TTE (N = 766)		THE (N = 766)		
	*N*	%	*N*	%	*P*	*N*	%	*N*	%	*P*
Intraoperative complications	116	4.8	59	4.9	0.870	45	5.9	36	4.7	0.368
Tube feeding	2120	88.0	1099	91.7	0.001	660	86.2	712	93.0	< 0.001
Type of tube feeding										
Jejunostomy	2006	83.3	1001	83.6	0.002	622	81.2	651	85.0	0.310
NJT	48	2.0	46	3.8		18	2.3	27	3.5	
Postoperative complications										
Thromboembolic	54	2.2	22	1.8	0.437	23	3.0	12	1.6	0.059
Neurologic/psychiatric	230	9.5	132	11.0	0.152	77	10.1	88	11.5	0.413
Infectious	116	4.8	91	7.6	0.001	47	6.1	66	8.6	0.099
Chyle leak	246	10.2	28	2.3	< 0.001	74	9.7	21	2.7	< 0.001
Gastro-intestinal	550	22.8	259	21.6	0.483	157	20.5	173	22.6	0.290
Urological	73	3.0	35	2.9	0.876	24	3.1	20	2.6	0.635
Pulmonary	805	33.	292	24.4	< 0.001	272	35.5	200	26.1	< 0.001
Cardiac	354	14.7	130	10.9	0.002	118	15.4	79	10.3	0.003
Anastomotic leakage	466	19.3	220	18.4	0.287	140	18.3	149	19.5	0.606
Recurrent nerve lesion	109	4.5	67	5.6	0.163	30	3.9	45	5.9	0.092
Any	1482	61.5	660	55.1	< 0.001	482	62.9	446	58.2	0.054
Reintervention										
Under GA	99	4.1	28	2.3	0.002	19	2.5	21	2.7	0.868
Radiological	248	10.3	63	5.3	< 0.001	81	10.6	43	5.6	0.001
Endoscopic	257	10.7	38	3.2	< 0.001	63	8.2	26	3.4	< 0.001
Surgical	370	15.4	115	9.6	< 0.001	113	14.8	71	9.3	0.002
Any	639	26.5	117	9.8	< 0.001	194	25.3	110	14.4	< 0.001
ICU stay*, median, days*	2	[1–5]	1	[1–4]	< 0.001	3	[1–6]	1	[1–3]	< 0.001
LOS*, median, days*	13	[9–21]	11	[9–16]	< 0.001	14	[10-23]	11	[9–16]	< 0.001
Readmittance	364	15.1	131	10.9	0.001	103	13.4	85	11.1	0.192
30-day/in-hospital mortality	88	3.7	30	2.5	0.082	31	4.0	13	1.7	0.009

Percentages might not add up to 100% due to rounding. Numbers between brackets depict the interquartile range. *GA* general anesthesia; *ICU* intensive care unit; *IQR* interquartile range; *LOS* length of stay; *MIS* minimally invasive surgery; *NA* not applicable; *NJT* nasojejunal tube

## Discussion

This study investigated the short-term outcomes of transthoracic and transhiatal esophagectomy for cancer in a nationwide propensity score matched analysis. The results show that a transthoracic approach provides a more thorough oncologic resection with a higher lymph node yield, at the cost of increased morbidity and short-term mortality. This is a population-based study, with all the variations in treatment this entails, giving a reflection of actual daily practice in the Netherlands. By utilizing a national database, we were able to study a much larger group of patients than would have otherwise been possible through a randomized, controlled trial. Furthermore, the present study reflects the results of the surgical treatment of esophageal cancer on a nationwide level compared with various publications, including only results from specialized tertiary centers. Our analysis therefore resembles real-world results more closely. However, this resemblance results in discrepancies compared with the guidelines: some patients with a mid-esophageal carcinoma were treated by THE, while the national guideline advises a transthoracic approach. Because this study also includes patients from smaller low-volume centers, the outcomes from our analysis also show, for example, higher anastomotic leakage and mortality rates and a lower lymph node yield compared with studies that only report outcomes of a single, specialized center or exclusively of high-volume (tertiary) centers.

The inclusion period of this study starts in 2011, which was the year of initiation of the DUCA. We know from earlier research that surgical care for esophageal cancer in the Netherlands has significantly evolved since 2011. The two largest changes in our country during the inclusion period of this study were the introduction of centralization of care with a minimum yearly hospital case-volume of 20 cases per year and the introduction of minimally invasive procedures.^7^^,^^11^ The process of implementing the minimum volume per hospital is still in progress. In the Netherlands in 2016, 22 hospitals performed esophagectomies for esophageal cancer.^12^ Five of these performed less than 20 resections in that year, three performed 20–29 resections, five performed 30-39 resections, and the remaining nine performed 40 or more resections in that year. This means that in 2016, five hospitals did not meet the minimum volume set in the national guidelines. The number of cases per surgeon is not recorded in the audit. Through the matching process we aimed to correct this potential bias by including the surgical approach and year of surgery as covariates. This means that at the start of the cohort, most minimally invasive procedures could not be matched due to the smaller volume of minimally invasive procedures, whereas in the more recent years of the cohort, many open esophagectomies could not be matched due to the smaller volume of open resections.

TTE provided a superior lymph node yield over THE, accompanied by a higher (y)pN stage compared with patients who underwent a THE. The results also show that 50% of the transhiatal resections had a lymph node yield lower than 14. In the case of a transthoracic resection, only 25% of patients had a lymph node yield below the national guideline of 15. Even though the number of positive nodes was equal in both groups, the question arises whether positive nodes have been missed in the lymph node dissection during transhiatal surgery. Because the cN stage was comparable between groups, although borderline matched (SMD 0.100), a selection bias causing patients with a higher cN to be more likely to undergo a TTE seems an unlikely explanation. The surgical community is divided on the value of an extended lymph node dissection after neoadjuvant chemoradiation. Noordman et al. concluded from their study with the CROSS cohort, that nCRT for esophageal adenocarcinomas might reduce the need for an extended lymphadenectomy, as can be performed with a transthoracic resection.^13^ A transthoracic resection was independently associated with a more favorable prognosis in the surgery alone group, whereas a TTE with additional nCRT was not. In addition to this, Kurokawa et al. prospectively investigated the distribution of lymph node metastases in gastroesophageal junction tumors and found that a limited lymph node resection could be sufficient in patients with tumors involving less than 4 cm of the esophagus.^14^ Another study investigating whether a subgroup of patients could benefit from conservative management following neoadjuvant therapy is currently ongoing in the Netherlands.^15^ On the contrary, a recent study found an association between an extensive lymph node dissection during esophagectomy and prolonged survival.^16^ Furthermore, a recent study by Raja et al. on post-neoadjuvant esophageal resection found that resecting up to 25 lymph nodes in ypN0 tumors or resecting up to 30 lymph nodes in ypN + tumors resulted in increased survival.^17^ The presence of positive nodes after nCRT has been associated with survival, which makes lymph node dissection essential for determining the prognosis.^18^ Additionally, TTE has been linked to a higher 5-year survival compared with THE in the case of positive nodes in the resection specimen, making the risk of potentially missed positive nodes after THE even more relevant.^5^

Our analysis showed that patients treated by a transthoracic esophagectomy experienced more chyle leakages, pulmonary, and cardiac complications. Additionally, they needed more reinterventions and had a longer ICU and hospital stay. The number of recurrent nerve lesions of any severity was comparable between TTE versus THE. The THE group received tube feeding more frequently, also after propensity score matching. This difference could be explained by differences in treatment protocols between hospitals. Unfortunately, information on hospital of treatment and treatment protocol is lacking in the DUCA database, so this theory cannot be confirmed by data in this study. The short-term mortality (in-hospital/30-day mortality) was significantly higher in the transthoracic group. The authors suspect that despite a comparable anastomotic leakage rate, the lower mortality in the THE group may be explained by less severe manifestations of leakage. As a result of reduced surgical pleural dissection, especially in the upper mediastinum, any leakage will likely result in limited mediastinal manifestations. This hypothesis is supported by earlier research showing a reduced incidence of intrathoracic complications of anastomotic leakage after a THE (27%) compared with a TTE with cervical anastomosis (44%) with similar incidence of anastomotic leakage between groups.^19^

Many publications regarding this subject suffer from bias since patients undergoing a THE generally differ significantly from those undergoing a TTE. Although some centers prefer one of these approaches for all their patients, most studies show evidence of selective allocation to the procedures based on preoperative condition, comorbidities of the patient, and cTNM staging. The current study shows that the known increased morbidity and mortality after a transthoracic approach for esophagectomy can be expected even in patients matched on baseline characteristics. A randomized trial comparing total gastrectomy via an abdominal-transhiatal approach versus a left thoracoabdominal approach found results similar to this study: more complications in the group with the extended approach.^20^ In addition, survival did not improve with the more aggressive approach in this randomized, controlled trial. Results however, cannot directly be extrapolated to the patients in this study, because this randomized, controlled trial compares gastrectomy for cardia and subcardia cancer and not esophagectomy for esophageal and gastroesophageal junction cancer.

The generalization of our results is reduced by the fact that we excluded hybrid procedures and nongastric-tube reconstructions after esophagectomy. Performing a subgroup analysis after propensity score matching is statistically unfeasible.^21^ We have explored the possibility of a 3-arm propensity score matching analysis (TTE with intrathoracic anastomosis, TTE with cervical anastomosis, and THE). However, because propensity score matching discarded nonmatched subjects, this led to very small groups no longer representative of the original cohort. In addition, because only the transthoracic group could be divided in an intrathoracic and cervical anastomosis group, correcting for this confounder was not possible. Because the audit does not disclose the hospital of origin of patients, we were unable to compare the two groups regarding the relation between hospital volume and complications, mortality, and pathology outcome. Because the results of this study may be influenced by the results of individual hospitals or by hospital volume, this is a limitation of the current study. In addition, evaluation of large cohorts of patients could lead to an inherent selection bias: the choice of the surgery type can be made based on experience, principle but also by necessity. Apart from this possible selection bias, propensity score matching does not correct for unknown confounders, and as such residual bias may be present. As stated in the *Methods* section, DUCA only registers outcomes during the first 30 postoperative days. Because of this, long-term outcomes are not available for analysis. Data regarding lymph node yield per region was only recently added to the audit, and subject to change, and therefore not included. Additionally, we do not have any information on location of suspicious lymph nodes, which could have led to a TTE for oncologic reasons, nor on information regarding surgical preference. Survival data are not part of the DUCA registration: this would have increased the value of the mortality analysis. The current study is nonetheless of great value because of the large number of patients included and correction for the often-reported selection bias through propensity score matching on baseline characteristics.

## Conclusions

Our analysis showed that, even after correction for baseline characteristics, a transthoracic approach provides a higher lymph node yield, at the cost of increased morbidity and short-term mortality. The lower lymph node yield after a transhiatal resection could indicate positive lymph nodes left in situ. Although results in high-volume centers and RCTs often are superior, these data reflect the national performance. We believe future research should investigate further whether long-term survival differs between a transthoracic and transhiatal resection in the era of (neo)adjuvant therapy, minimally invasive surgery, and increasingly centralized care.
